# Early menopause and weight loss are significant factors associated with risk of future fracture in middle-aged women

**DOI:** 10.1186/s12891-022-05744-5

**Published:** 2022-08-16

**Authors:** Louise Moberg, Viktor Hamrefors, Artur Fedorowski, Cecilia Rogmark

**Affiliations:** 1 Department of Obstetrics and Gynaecology, Skåne University Hospital, Klinikgatan 12, 221 85 Lund, Sweden; 2 Department of Clinical Sciences, Lund University, Lund, Sweden; 3 Department of Clinical Sciences, Lund University, Malmö, Sweden; 4 Department of Internal Medicine, Lund, Skåne University Hospital, Malmö, Sweden; 5 Department of Cardiology, Karolinska University Hospital, Stockholm, Sweden; 6 Department of Orthopaedics, Skåne University Hospital, Malmö, Sweden

**Keywords:** Aging, Fracture prevention, General population studies, Menopause, Weight change

## Abstract

**Background:**

To identify factors related to reproductive history and weight change associated with first incident fracture in middle-aged women.

**Methods:**

In total, 18,326 women from the Malmö Diet and Cancer study were included in this prospective population-based cohort study. Participants were included 1991–1996 and followed to 2016. Using data from the National Patient Registry, linked with every participants’ unique personal identification number, any first fracture affecting spine, thoracic cage, upper and lower extremities was identified. The association of baseline factors with incident fracture risk was analyzed using Cox regression models.

**Results:**

For participating women, median age 56.0 years, the multivariable Cox regression analysis observed that early menopause (40–44 years) (hazard ratio (HR) 1.14, 95% confidence interval (CI) 1.03–1.27) but not premature menopause < 40 years (HR 1.06, 95% CI 0.91–1.24) was associated with future fracture risk. Self-reported weight loss since age 20 was also associated with future fracture risk (HR 1.39, 95% CI 1.17–1.65) whereas a daily alcohol consumption in the third quartile (5.36–11.42 g/day) compared to the lowest quartile (0–0.80 g/day) was associated with decreased future fracture risk (HR 0.88, 95% CI 0.81–0.96). The multivariable Cox regression analysis also observed that increasing age and weight at baseline, current smoking, a positive history of previous fracture and family history of fractures were associated with increased fracture risk whereas an increasing BMI was associated with a decreased fracture risk. No association to parity or period of lactation was observed nor ever-use of oral contraceptives and menopausal hormone therapy.

**Conclusion:**

This study shows that early menopause between 40 to 45 years and self-reported weight loss since age of 20 are relevant factors associated with increased fracture risk in middle-aged women. These factors were independent of traditional predictors of fracture risk among women and may be considered in preventive initiatives.

**Trial registration:**

Clinicaltrials.gov with identifier: NCT04151732, since Nov 5th 2018.

## Background

With rising longevity, an increase in the number of fractures is observed [1]. Besides increasing age, heredity for osteoporosis, smoking and previous fracture, being female is a risk factor for sustaining fractures [2]. To prevent fractures in women particular sex-specific risk factors need to be identified [3, 4]. While some reproductive factors influencing the female fracture risk are more established, e.g. the association between both later menarche and earlier menopause and increased fracture risk [5], results regarding parity and breastfeeding are diverging. Studies on association of parity and risk of fracture in general and hip fracture in particular have reported both increased fracture risk with increasing parity [6], no association with fracture risk [7–9] and decreased fracture risk with increasing parity [5, 10–12]. Lactation has also been studied in association with fracture risk and both increased risk with increased duration of breastfeeding [5], no association [7, 8] and decreased risk has been shown [9]. However, these divergences in observed associations may be due to differences in study design e.g. methodology: meta-analysis [12], case-control study [6], or observational study [7]; inclusion criteria e.g. 42–53 years at inclusion [8], or 50–94 years at inclusion [9]; study size varying from 200 postmenopausal women [6] to 93,676 postmenopausal women [7]; as well as study populations from different regions in the world e.g. Taiwan [13] and Norway [9]. Menopausal hormone therapy has been demonstrated to have a bone-protective effect reducing the number of fractures [14]. Treatment with estrogens is also recommended for women with premature or early menopause [15].

Weight changes during adult life have been studied in women with somewhat different results. A large study from the Women’s Health Initiative including approximately 120,000 postmenopausal women mean age 63.3 years at baseline observed a differential effect of weight loss and weight gain on site of fracture during follow-up e.g. weight gain was associated with higher incidence rates of hip fracture whereas weight loss increased risk of lower limb fractures compared to women with stable weight [16]. In a study of women with a mean age of 68 years, weight loss was associated with increased fracture risk and mortality during a 20-year follow-up [17].

So, the understanding of reproductive factors and weight changes in this aspect is limited. Using a large prospective cohort with self-reported baseline data gives an opportunity to deepen the knowledge in the field. Thus, we aimed to identify factors related to reproductive history and weight change associated with first incident fracture in middle-aged women.

## Methods

### Participants

The Malmö Diet and Cancer Study (MDCS) study is a population-based, prospective cohort study that has been described in detail previously [18]. The overall aim of MDCS was to investigate the relationship between diet at baseline and cancer incidence during follow-up [19]. In short, all inhabitants in the city of Malmö born 1923 to 1950 (*n* = 74,138) were invited to participate in the study, between 1991 and 1996. A total of 30,446 inhabitants accepted the invitation, of which 18,326 (60.2%) were women. Invitations were sent out by postal mail and via advertisements in newspapers and public places [18]. The baseline study entails a detailed questionnaire and a physical examination including e.g. measurement of height (cm), weight (kg), measurements of waist and hip circumference (cm) [20]. At the initial visit the participants were firstly given information about the study in a group of participants [19]. Secondly, an individual measurement of anthropometric variables was performed one participant at a time [19]. Two weeks later the participant come back with their filled-in questionnaire and the questionnaires were gone through with the study staff [21]. A previous study has shown that more non-participants than participants died during follow-up and more were diagnosed with cancer, hence there could be an element of “the healthy cohort effect” [22].

### Descriptive variables

From the examination at baseline measurement of weight (kg) and height (cm) was obtained. Body mass index (BMI) (kg/m^2^) was calculated using weight and height data. Using the measurements of circumference at the waist (cm) and the hip (cm) the individual waist-hip-ratio was calculated. Self-reported variables were extracted from the questionnaire and reported as follows. Current smoking (yes) was composed of the response options: “yes, I smoke regularly” and “yes, I smoke occasionally” compared to “no”. Alcohol consumption was calculated in g/day from reported intake of beer, wine and spirits during a 7-day diet report and was further divided into quartiles (quartile 1 (0–0.80 g/day), quartile 2 (0.81–5.35 g/day), quartile 3 (5.36–11.42 g/day), and quartile 4 (> 11.43 g/day)). Weight at 20 years of age is reported in kilograms (kg). The weight change from 20 years of age was calculated by subtracting the self-reported weight at 20 years from the measured weight at baseline examination. “Weight change from 20 years of age” was constructed of 4 alternatives: “it has been the same”, “gradually increased”, “gradually decreased” and “my weight has varied”. Living alone (yes/no), heavy work (yes/no) and highest level of education (“did not complete elementary school”, “elementary and junior school (6-10 years)” and “advanced level and university (> 11 years)”) were all self-reported.

The physical activity score has previously been described in detail and validated in a subset of the cohort [23]. In short, the questionnaire contained 18 questions regarding activities during the year and a summary score was calculated by combining the intensity factor of each activity with the time of each activity (these questions were adapted and modified from the Minnesota leisure-time physical activity questionnaire) [23]. A higher score reflects a higher level of physical activity.

The time of follow-up (years) was calculated from individual inclusion in the study until date of first incident fracture, death, immigration or end of follow-up (31st of December 2016) whichever came first.

### Reproductive variables

Included reproductive variables were all self-reported and answers were grouped into different categories. “Age at menarche” was grouped as: < 11, 12–15 and > 16 years of age. “Age at menopause” was grouped as: < 40, 40–44, 45–54 and > 55 years. Participating women were both premenopausal and postmenopausal. The reproductive period was calculated by subtracting the “age at menarche” from the “age at menopause”. The “number of born children” were grouped as: no children, 1–2 children and > 3 children. “Total duration of breast-feeding” was calculated by adding up all individual periods of breast-feeding reported per child and was grouped as: no breast-feeding (including both women never giving birth and women giving birth but never breastfeeding), 1–6, 7–18 and > 19 months of total breast-feeding. “Age at first delivery” was calculated from the year of first birth and grouped as: < 19, 20–34 and > 35 years of age. “Ever use of menopausal hormone therapy” was grouped as yes/no. “Duration of use of menopausal hormone therapy” (years) was included as a continuous variable. “Miscarriages > 3” was extracted from number of reported miscarriages and grouped as yes/no for the occurrence of 3 or more miscarriages. The question regarding previous surgery with hysterectomy and/or unilateral or bilateral salpingoophorectomy was used to create the variable “previous hysterectomy and/or salpingoophorectomy” and grouped as yes/no. “Ever use of oral contraceptives” was reported as previous or current use, grouped as yes/no. The self-reported total duration of use of oral contraceptives was used to calculate “total use of oral contraceptives” < 10, 11–19 and > 20 years. “Start use of oral contraceptives at age” was included as a continuous variable.

### Fracture data

Data regarding first incident fracture were obtained by crosslinking of every participant’s unique Swedish personal identification number with the Swedish National Patient Registry. The validity of the inpatient part of the Swedish National Patient register has been studied and is considered to be high with a positive predictive value of 85–95% of diagnoses overall [24]. For fractures, the registry has been validated for hip fractures compared to the Swedish Hip Registry (RIKSHÖFT), which is another national Swedish registry only including hip fractures, and the coverage has been considered excellent [25].

If several fractures occurred at the same event, this was considered as one incident fracture event. A fracture was included if occurring after the participant’s inclusion in the study and before the end of follow-up (31st of December 2016). The registry contains in-patient diagnoses since 1970 for southern Sweden and national coverage since 1987. For out-patient diagnoses coverage is from 2001 and onwards i.e. fractures occurring earlier were included if they demanded in-hospital care.

As the definition of “fragility fracture” or “osteoporotic fracture” has changed during time and no unanimous definition prevails, we included all fracture sites except skull and face as currently suggested in the literature [26]. The current study defines outcomes as fractures affecting the spine and the thoracic cage (S12.x, S22.x, S32.x), arms, shoulders and hands (S42.x, S52.x and S62.x), the pelvis (S32.x), as well as the hip, femur (S72.x) and lower leg (S82.x). In addition, codes regarding spontaneous fractures were also sought (M48.4, M84.3, and M96.6 (M97 is not used in Sweden)). The fracture codes are defined by the ICD-10 system, used in Sweden since 1997. For fractures occurring before that, the ICD-9 was used. This was relevant for a total of 503 fractures were included in the study. Occurrence of previous fracture before baseline inclusion for the participants was also obtained from the Swedish National Patient Registry. Family history for fractures was self-reported as any fracture occurrence in either father, mother or sibling after the age of 50 years. For this variable, a missing-value (*n* = 9374) or the answer “do not know” was imputed as a no.

### Statistical analysis

All continuous variables were tested for normality with the one-sided Kolmogorov Smirnov test and all were non-parametric. Hence, the Mann-Whitney U test was used for comparisons of continuous data between the groups of women with and without incident fracture during follow-up. Non-parametric data are presented as median (value of 25th quartile and 75th quartile). For grouped data, the Chi-squared test was used.

For all variables with a significant difference between women with and without a first incident fracture an initial Cox regression analysis adjusted for age, BMI and previous fracture (basic model) was performed for each variable, with the exception of kg weight change from 20 years of age, as it was included with the question “How has your weight changed from 20 years of age?”. A further multivariable-adjusted analysis was performed including all variables with a significant impact (*p* < 0.05) on fracture risk from the basic model analysis including adjustment variables (age, BMI and previous fracture). Results are presented as hazard ratios (HR) with a 95% confidence interval (CI). For categorical variables, the proportional hazards assumption was assessed by visual inspection of survival curves. The proportionality assumption was fulfilled for all main variables of the current study. Smoking, which was used as a covariate in the multivariable-adjusted analyses, was a significant predictor of fractures only after adjustment for age. Thus, the KM curves for smoking were nearly identical for smokers and non-smokers.

All analyses were performed using IBM SPSS Statistics version 25 (SPSS Inc., Chicago, IL, USA). All tests were two-sided and a *p*-value of < 0.05 was considered statistically significant.

## Results

Of the 18,326 women that was included in the analyses, 6023 suffered a first incident fracture during a mean follow-up of 13.8 (9.3–18.2) years.

Regarding baseline characteristics women having one or several fractures were older, had lower BMI and weight, drank less alcohol and fewer were current smokers compared to women without fracture(s) during follow-up. In addition, women in fracture group more often have had a previous fracture, had a positive family history of fractures, more often lived alone, and had lower education level. When comparing the actual weight in kilograms at 20 years of age, there was no significant difference between women with and without fracture during follow-up, but more women in fracture group stated that they had reduced their weight from 20 years of age (Table 1).

**Table 1:** Descriptive baseline data for all women and women with and without incident fracture

				Incident fracture		
			No		Yes	
			*n* = 12,303		*n* = 6023	
	n^a^		median (Q2-Q4^b^)		median (Q2-Q4^b^)	*p*-value
Age at baseline	18,323	(years)	55.2. (49.5–62.4)		60.0 (52.0–65.5)	**< 0.001**
Body mass index (BMI)	18,293	(kg/m^2^)	24.8 (22.5–27.9)		24.7 (22.5–27.6)	**0.004**
Weight	18,293	(kg)	67.0 (60.0–75.0)		66.0 (60.0–74.0)	**0.005**
Height	18,294	(cm)	163.0 (159.0–168.0)		164.0 (159.0–168.0)	0.586
Weight at 20 years of age	15,143	(kg)	55.0 (50.0–60.0)		55.0 (50.0–60.0)	0.152
Waist-hip-ratio	18,285	(ratio)	0.79 (0.76–0.83)		0.79 (0.76–0.83)	0.158
Alcohol	17,100	(g/day)	5.6 (0.8–11.7)		4.8 (0.6–11.0)	**< 0.001**
Physical activity score	17,205		6720.0 (4000.0–10,365.0)		6730.0 (3877.0–10,575.0)	0.754
			n (%)		n (%)	
Current smokers	17,319	(yes)	3336 (28.7)		1526 (26.8)	**0.008**
Previous fracture	18,326	(yes)	299 (2.4)		277 (4.6)	**< 0.001**
Family history of fracture	18,326	(yes)	2199 (17.9)		1392 (23.1)	**< 0.001**
Living alone	17,315	(yes)	3149 (27.1)		1750 (30.7)	**< 0.001**
Heavy work	16,873	(yes)	1455 (12.8)		732 (13.2)	0.469
Highest level of education	17,278					**< 0.001**
Completed elementary school		(no)	96 (0.8)		50 (0.8)	
Elementary and junior school (6–10 years)		(yes)	7814 (67.4)		4068 (71.5)	
Advanced level and university (> 11 years)		(yes)	3677 (31.7)		1573 (27.6)	
Self-reported weight change since 20 years of age	17,288					**< 0.001**
It has been the same		(yes)	1452 (12.5)		744 (13.1)	
Gradually increased		(yes)	6787 (58.5)		3216 (69.5)	
Gradually decreased		(yes)	280 (2.4)		210 (3.7)	
My weight has varied		(yes)	3075 (26.5)		1524 (26.8)	
			median (Q2-Q4^b^)		median (Q2-Q4^b^)	
Weight changes in kilograms for groups of self-reported weight change since 20 years of age	12,150					**0.001**
It has been the same	1641	(kg)	3.0 (1.0–7.0)		3.0 (0.0–6.0)	
Gradually increased	7116	(kg)	14.0 (9.0–20.0)		14.0 (9.0–20.0)	
Gradually decreased	358	(kg)	−4.0 (−8.0- -0.5)		−5.0 (−8.0- -2.0)	
My weight has varied	3035	(kg)	9.0 (4.0–15.0)		9.0 (4.0–15.0)	
Overall weight change since 20 years of age	12,163	(kg)	11.0 (6.0–18.0)		11.0 (5.0–17.0)	**< 0.001**

^a^the number of valid answers for every question, used for calculating percentages

^b^values of quartile 2 and 4

As for reproductive history, a higher proportion of women in the fracture group were > 16 years of age at menarche and > 55 years at menopause, more often had > 3 children, fewer gave birth at ages < 19 years or > 35 years and had a tendency toward longer periods of lactation. They used oral contraceptives, but less frequently and started at an older age than women without a fracture. There was also a tendency towards shorter duration of oral contraceptives use. There was no difference in the number of users of menopausal hormone therapy, women who had had > 3 miscarriages or more, and no difference in the number of women who had had a hysterectomy and/or salpingoophorectomy (Table 2). A sub-analysis was performed comparing the number of ever-users of menopausal hormone therapy between women < 40 at age of menopause (45.8%) compared to women 40–44 (43.2%) but the difference did not reach statistical significance (*p* = 0.398).

**Table 2:** Reproductive data reported at baseline for all women and women with and without incident fracture

				Incident fracture		
			No		Yes	*p*-value
			*n* = 12,303		*n* = 6023	
	n^a^					
Age at menarche	17,189		n (%)		n (%)	**< 0.001**
< 11		(years)	826 (7.2)		335 (5.9)	
12–15		(years)	9731 (84.4)		4774 (84.3)	
> 16		(years)	968 (8.4)		555 (9.8)	
Age at menopause	12,468					**0.029**
< 40		(years)	358 (4.5)		172 (3.8)	
40–44		(years)	776 (9.8)		443 (9.8)	
45–54		(years)	6085 (76.6)		3438 (76.0)	
> 55		(years)	723 (9.1)		473 (10.5)	
Number of born children	17,028					**0.01**
no children			1466 (12.8)		756 (13.5)	
1–2 children			7347 (64.2)		3455 (61.8)	
≥ 3 children			2628 (23.0)		1376 (24.6)	
Total duration of lactation	18,326					
no lactation		(months)	3202 (26.0)		1623 (26.9)	**0.002**
1–6		(months)	3918 (31.8)		1752 (29.1)	
7–18		(months)	4180 (34.0)		2149 (35.7)	
≥ 19		(months)	1003 (8.2)		499 (8.3)	
Age at first delivery	14,794					**0.007**
≤ 19		(years)	1295 (13.0)		542 (11.2)	
20–34		(years)	8318 (83.5)		4121 (85.4)	
≥ 35		(years)	354 (3.6)		164 (3.4)	
Ever use of menopausal hormone therapy	7441	(yes)	1336 (26.4)		663 (27.8)	0.229
Hysterectomy and/or salpingoophorectomy	7457	(yes)	769 (15.2)		396 (16.6)	0.125
Miscarriages > 3	3892	(yes)	249 (9.5)		139 (10.9)	0.157
Ever use of oral contraceptives	17,306	(yes)	6049 (49.2)		2441 (40.5)	**< 0.001**
Total use of oral contraceptives	8206					**0.027**
< 10		(years)	3676 (62.8)		1525 (64.9)	
11–19		(years)	1162 (19.8)		471 (20.1)	
> 20		(years)	1020 (17.4)		352 (15.0)	
			median (Q2-Q4^b^)		median (Q2-Q4^b^)	
Start use of oral contraceptives at age	8323	(age)	25.0 (21.0–31.0)		27.0 (22.0–34.0)	**< 0.001**
Reproductive period	12,398	(years)	36.0 (33.0–39.0)		36.0 (33.0–39.0)	0.088
Total use of menopausal hormone therapy	1763	(years)	3.0 (2.0–6.0)		3.0 (2.0–7.8)	**0.004**

^a^the number of valid answers for every question, used for calculating percentages

^b^ values of quartile 2 and 4

The results of the basic and multivariable-adjusted models’ Cox-regression analyses are shown in Tables 3 and 4, respectively. *Age at menopause* between 40 and 44, i.e. an early menopause, was associated with an increased future fracture risk of 14% in both basic and multivariable-adjusted models compared to menopause at 45–54 years of age. Both *parity* and *lactation* showed an association with future fracture(s) in the basic model but not in the multivariable-adjusted analysis. Having had 1–2 children decreased fracture risk with 8% compared to not having given birth. Likewise, in the basic model, all analysed periods of lactation were associated with a 9–11% lower risk compared to women reporting never to have breastfed.

**Table 3:** Individual background factors and risk of first incident fracture

			HR^a^	95% CI^b^	*p*-value
		n^c^			
Age at baseline	(years)	18,293	1.06	1.06–1.06	**< 0.001**
Body mass index (BMI)	(kg/m^2^)	18,293	0.98	0.97–0.98	**< 0.001**
Previous fracture	(yes)	18,293	1.80	1.59–2.03	**< 0.001**
Current smoking	(yes)	17,290	1.14	1.08–1.21	**< 0.001**
Living alone	(yes)	17,286	1.07	1.01–1.13	**0.024**
Family history of fractures	(yes)	18,293	1.16	1.09–1.23	**< 0.001**
Weight at baseline	(kg)	18,293	1.01	1.01–1.02	**< 0.001**
Highest level of education		17,249			
Completed elementary school			Reference		
Elementary and junior school (6–10 years)			0.88	0.67–1.16	0.371
Advanced level and university (> 11 years)			0.90	0.68–1.19	0.444
Weight change since 20 years of age		17,259			
It has been the same			Reference		
Gradually increased			1.03	0.94–1.12	0.565
Gradually decreased			1.38	1.19–1.61	**< 0.001**
My weight has varied			1.10	1.01–1.21	**0.032**
Alcohol	(g/day)	17,072			
Quartile 1 (0–0.80)			Reference		
Quartile 2 (0.81–5.35)			0.94	0.87–1.01	0.079
Quartile 3 (5.36–11.42)			0.87	0.81–0.94	**< 0.001**
Quartile 4 (> 11.43)			0.94	0.87–1.02	0.123
Age at menarche	(years)	17,161			
< 11			0.97	0.87–1.09	0.642
12–15			Reference		
> 16			1.08	0.99–1.18	0.084
Number of born children		16,999			
no children			Reference		
1–2 children			0.92	0.85–0.995	**0.038**
≥ 3 children			0.97	0.88–1.06	0.428
Total duration of lactation	(months)	18,293			
no lactation			Reference		
1–6			0.89	0.84–0.96	**0.001**
7–18			0.91	0.86–0.97	**0.006**
≥ 19			0.89	0.81–0.99	**0.029**
Age at first delivery	(years)	14,777			
≤ 19			1.002	0.92–1.10	0.973
20–34			Reference		
≥ 35			0.99	0.85–1.16	0.886
Ever use of oral contraceptives	(yes)	17,277	0.96	0.91–1.02	0.227
Total use of oral contraceptives	(years)	8199			
< 10			Reference		
11–19			0.95	0.85–1.05	0.302
> 20			0.90	0.80–1.01	0.067
Start use of oral contraceptives at age		8316	0.99	0.98–1.001	0.089
Age at menopause	(years)	12,444			
< 40			1.05	0.90–1.22	0.544
40–44			1.14	1.04–1.26	**0.008**
45–54			Reference		
> 55			0.96	0.87–1.06	0.384
Total use of menopausal hormone therapy	(years)	1761	0.99	0.97–1.01	0.209

Analyses were adjusted for age, body mass index (BMI) and history of previous fracture in the Cox regression models

^a^Hazard Ratios

^b^Confidence Interval

^c^the number of valid answers for every variable

**Table 4:** Individual background factors and risk of first incident fracture in the multivariable-adjusted model

	(*n* = 12,018^a^)	HR^b^	95% CI^c^	*p*-value
Age at baseline	(years)	1.07	1.06–1.07	**< 0.001**
Body mass index (BMI)	(kg/m^2^)	0.95	0.94–0.97	**< 0.001**
Previous fracture	(yes)	1.64	1.43–1.88	**< 0.001**
Current smoking	(yes)	1.12	1.04–1.20	**0.003**
Living alone	(yes)	1.03	0.96–1.10	0.44
Family history of fractures	(yes)	1.12	1.05–1.20	**0.001**
Weight at baseline	(kg)	1.01	1.01–1.02	**0.001**
Weight change since 20 years of age				
It has been the same		Reference		
Gradually increased		0.99	0.90–1.10	0.897
Gradually decreased		1.39	1.17–1.65	**< 0.001**
My weight has varied		1.07	0.96–1.19	0.22
Alcohol	(g/day)			
Quartile 1 (0–0.80)		Reference		
Quartile 2 (0.81–5.35)		0.96	0.88–1.04	0.285
Quartile 3 (5.36–11.42)		0.88	0.81–0.96	**0.002**
Quartile 4 (> 11.43)		0.96	0.88–1.05	0.346
Number of born children				
no children		Reference		
1–2 children		0.99	0.86–1.14	0.902
≥ 3 children		1.02	0.88–1.19	0.782
Total duration of lactation	(months)			
no lactation		Reference		
1–6		0.96	0.85–1.09	0.554
7–18		0.97	0.86–1.09	0.586
≥ 19		0.94	0.80–1.10	0.434
Age at menopause	(years)			
< 40		1.06	0.91–1.24	0.439
40–44		1.14	1.03–1.27	**0.009**
45–54		Reference		
> 55		0.97	0.88–1.07	0.501

Individual background factors in relation to first incident fracture in a multivariable Cox regression model

^a^The multivariate model includes 12,018 women. This is due to missing-data for some variables, which is the greatest for age at menopause (*n* = 5855)

^b^Hazard Ratios

^c^Confidence Interval

Weight and BMI at baseline, and weight decrease since the age of 20 years were associated with fractures in both analyses. A greater weight at baseline increased future fracture risk with 1% per kg. A self-reported weight loss since the age of 20 years increased future fracture risk by 38–39%. A lower BMI increased future fracture risk with 5% per unit in the multivariable-adjusted model.

*Current smoking* was associated with future fracture risk in both basic model and the multivariable-adjusted model. The risk increased with 12–14% depending on analysis model. A daily *alcohol consumption* in the 3rd quartile, was associated with a fracture risk reduction of 12–13% was compared to those in the lowest quartile of alcohol consumption depending on analysis model. *Other background factors* such as higher age, a positive family history of fractures and previous fracture before inclusion in the study were associated with fracture risk. A previous fracture had the greatest increased risk of future fracture of 64%.

## Discussion

In this population-based cohort, with self-reported data at baseline, weight loss since 20 years of age and early menopause were associated with a higher risk of incident fracture in middle-aged women. Moreover, our results do not support the suggestion that parity, lactation, ever-use of oral contraceptives or menopausal hormone therapy is associated with future fractures.

Premature menopause or premature ovarian insufficiency and early menopause occurs in every tenth woman [27]. For women with a premature or early menopause, treatment with menopausal hormone therapy up until at least normal menopausal age is recommended, to prevent the detrimental effects of estrogen deficiency on e.g. the skeleton and the cardiovascular system [27]. In this study, women with menopause occurring under the age of 40 did not have an association with first incident fracture whereas women with menopause between 40 and 44 years of age did. It could be that the clinical interest has been focused on the youngest women experiencing premature menopause. There was a non-significant trend in this material that fewer women with menopause at ages 40–44 (43.2%) were ever-users of menopausal hormone therapy than for women < 40 at age of menopause (45.8%). It may also be that women with menopause between 40 and 44 years of age did not seek medical attention, considering it normal. Similar observations were made in a recent meta-analysis and systematic review, including 18 studies and 462,393 postmenopausal women, that premature ovarian insufficiency (< 40 years) was not associated with an increased fracture risk whereas early menopause (40–45 years) increased the fracture risk [28]. Being underweight may also trigger an earlier menopause hence the observed findings may be multifactorial [27].

For women in this cohort, an increased risk of future fracture was observed with both lower BMI and higher weight at baseline. One can assume that weight, height and BMI can interfere with fracture risk via complex pathways and the relationship may be different per fracture site [29]. Taller women in particular have a higher risk of hip fracture [30]. A self-reported weight loss from 20 years of age was associated with an almost 40% higher future fracture risk. The increased risk may be caused by medical conditions, side-effects of medications or illnesses that in themselves carry an increased fracture risk, but also voluntary weight loss in obese, elderly individuals has been shown to be associated with a loss of bone mineral density [31]. In a study of elderly women, weight loss was associated with increased fracture risk and mortality during a 20-year follow-up from a mean age of 68 years to a mean age of 88 years [17].

Oral contraceptives have been shown to affect bone mineral density, but, notably, fracture was not the endpoint [32]. The evidence is difficult to entangle: use of oral contraceptives has been associated with smaller gains of bone mass in adolescents [33], and a recent study observed an 3–6% increased fracture risk with > 1 year of oral contraceptive use [5]. On the other hand, decreased risk of hip fracture has been described after use of oral contraceptives in late reproductive years [34]. When used in the premenopausal years it may not affect fracture risk [35]. Our crude data described fewer ever users of oral contraceptives in women with fracture(s), starting their oral contraceptives use at an older age and using them for a shorter period than women without fracture. None of these factors were, however, associated with fracture risk in the basic model analysis.

We found no observed difference in the number of ever-users of menopausal hormone therapy among subjects with and without incident fractures, hence the variable was not further explored. Menopausal hormone therapy has been shown to have a protective effect on bone and reduce fracture risk [14]. The inclusion period for the current study, 1991 to 1996, was at a time when menopausal hormone therapy was popular. Still our rate of 11% users is low compared to another Swedish study showing a marked increase in use of menopausal hormone therapy from 13% in 1992 to 31% in 1998 [36]. In addition, our participants had a median age of 57 years at inclusion. Several may have passed the age where menopausal hormone therapy is mainly prescribed. However, it is possible that some women, who at baseline were not using menopausal hormone therapy, may have been prescribed this at a later stage after inclusion and this may affect the results. It is also not known if there were any iatrogenic reasons for premature or early menopause e.g. breast cancer disabling the use of menopausal hormone therapy that may have affected the results. We found a protective effect on fracture risk for women who had 1–2 children compared to nullipara, in the basic model analysis, but the results did not remain significant in the multivariable-adjusted model. A recent Swedish study observed a decreased risk for hip fractures in individuals with children compared to those without children. The effect was most pronounced in individuals with 2–3 children similar to the results of our study [10]. Another Swedish study on postmenopausal women aged 50–81 years observed a 10% decrease in hip fracture risk per born child and attributed this to the weight gain associated with pregnancy [37]. Breastfeeding has been widely discussed regarding the effect on the skeleton and if that effect is permanent and associated with fracture risk [5, 7–9]. Our finding, that women who breastfed for more than 1 month had a 9–11% decreased risk of first incident fracture, are similar to the results reported by Crandall et al. in a study of 93,676 postmenopausal where breastfeeding for at least 1 month, compared to no breastfeeding, was associated with a significant decreased hip fracture risk of 16% [7]. Our finding did however, not remain significant in the multivariable-adjusted model analysis. A Korean study on 1,272,115 postmenopausal women, observed an increased risk of any fracture after more than 12 months of breastfeeding but a decreased risk of hip fracture [5]. As results on lactation and fracture risk are diverging further studies are warranted.

As this is an observational study, there are several limitations that may affect the validity of our findings. Since subjects were not randomly assigned to participation there is the risk of “the healthy cohort effect”, well known in epidemiological studies and in the current cohort [22]. Causal associations cannot be conclusively drawn from the results. Many of the variables are self-reported with risk for bias concerning some questions e.g. alcohol consumption. Weight change since 20 years of age is also self-reported which may be considered as a limitation of this variable. It is a qualitive nominal variable limited by the fact that it is the participants’ interpretation of its weight and weight change since the age of 20 years that is reported. Ideally, there should have been measurements of weight performed at repeated intervals to give more certain data regarding individual weight change, but such a longitudinal design is impracticable. As the results for the calculated weight change in kilograms for the different response groups is congruent with the respective response group (i.e. in the group stating a decreased weight the calculated median weight change was − 4 kg and − 5 kg for women without and with incident fracture during follow-up), we regard the question to reflect the overall weight trajectory for the participants. The findings warrant further studies with long term follow-up of weight, with repeated measurements, to further elucidate weight change and its association with fracture risk. For some socioeconomic factors, it is also difficult to separate the individual effect as they may be closely related. Further, the inclusion in the study was between 1991 and 1996 and hence it is not certain that the current results are fully representative for middle-aged women today. The study does not have bone density data for the participants, meaning that previous fracture served as the surrogate variable for this. The multivariable analysis includes only 12,018 of the women in the study due to missing-values mainly for age at menopause. This could be due to natural physiological reasons as some women may not have experienced menopause at the time of the baseline questionnaire or that women did not want to answer the question in the questionnaire. The questionnaire from the baseline inclusion in the study lacks information on the age of the women at the time of hysterectomy and/or salpingoophorectomy, and whether the salpingoophorectomy was unilateral or bilateral. This limits the analysis of the variable in respect to hormonal changes and associated fracture risk. This is also true when it comes to the use of menopausal hormone therapy that information is lacking regarding when this use occurred. Regarding lactation and breastfeeding, these two variables are very closely linked and it is difficult to separate the individual effect of both variables on associated fracture risk. Finally, the current study did not consider any potential interventions that may have been done during follow-up. Thus, we cannot exclude that such interventions may have influenced the results.

The main clinical implications of our results are that women with an early menopause should be informed regarding the potential increased fracture risk. Adequate treatment should be initiated if possible. Screening for additional fracture risk factors and corresponding interventions in women with an early menopause are likely to be important.

## Conclusions

In conclusion, early, but not premature, menopause and weight loss since age of 20 is associated with increased fracture risk in middle-aged women. Early menopause should not be considered only as a normal variant and treated lightly when it comes to the effects on fracture risk, but should be rather seen as an additional risk factor. We suggest that women with an early menopause and/or with gradual weight loss should be screened for additional fracture risk factors and adequate interventions should be considered.

## Data Availability

Due to the conditions of the ethics review board approval, a public repository cannot be used. Anonymized data are available upon reasonable request to the corresponding author.
